# Molecular and Functional Characterisation of a Novel Intragenic 12q24.21 Deletion Resulting in *MED13L* Haploinsufficiency Syndrome

**DOI:** 10.3390/medicina59071225

**Published:** 2023-06-29

**Authors:** Evelina Siavrienė, Gunda Petraitytė, Violeta Mikštienė, Živilė Maldžienė, Aušra Sasnauskienė, Vilmantė Žitkutė, Laima Ambrozaitytė, Tautvydas Rančelis, Algirdas Utkus, Vaidutis Kučinskas, Eglė Preikšaitienė

**Affiliations:** 1 Department of Human and Medical Genetics, Institute of Biomedical Sciences, Faculty of Medicine, Vilnius University, 08410 Vilnius, Lithuaniavaidutis.kucinskas@mf.vu.lt (V.K.);; 2 Department of Biochemistry and Molecular Biology, Institute of Biosciences, Life Sciences Centre, Vilnius University, 10257 Vilnius, Lithuania

**Keywords:** *MED13L* haploinsufficiency syndrome, intellectual disability, congenital anomalies, molecular and functional analysis, CRISPR-Cas9

## Abstract

*Background and Objectives:* Heterozygous pathogenic variants in the *MED13L* gene cause impaired intellectual development and distinctive facial features with or without cardiac defects (MIM #616789). This complex neurodevelopmental disorder is characterised by various phenotypic features, including plagiocephaly, strabismus, clubfoot, poor speech, and developmental delay. The aim of this study was to evaluate the clinical significance and consequences of a novel heterozygous intragenic *MED13L* deletion in a proband with clinical features of a *MED13L*-related disorder through extensive clinical, molecular, and functional characterisation. *Materials and Methods:* Combined comparative genomic hybridisation and single-nucleotide polymorphism array (SNP-CGH) was used to identify the changes in the proband’s gDNA sequence (DECIPHER #430183). Intragenic *MED13L* deletion was specified via quantitative polymerase chain reaction (qPCR) and Sanger sequencing of the proband’s cDNA sample. Western blot and bioinformatics analyses were used to investigate the consequences of this copy number variant (CNV) at the protein level. CRISPR-Cas9 technology was used for a *MED13L*-gene-silencing experiment in a culture of the control individual’s skin fibroblasts. After the *MED13L*-gene-editing experiment, subsequent functional fibroblast culture analyses were performed. *Results:* The analysis of the proband’s cDNA sample allowed for specifying the regions of the breakpoints and identifying the heterozygous deletion that spanned exons 3 to 10 of *MED13L*, which has not been reported previously. In silico, the deletion was predicted to result in a truncated protein NP_056150.1:p.(Val104Glyfs*5), partly altering the Med13_N domain and losing the MedPIWI and Med13_C domains. After *MED13L* gene editing was performed, reduced cell viability; an accelerated aging process; and inhibition of the *RB1*, *E2F1*, and *CCNC* gene expression were found to exist. *Conclusions:* Based on these findings, heterozygous intragenic 12q24.21 deletion in the affected individual resulted in *MED13L* haploinsufficiency due to the premature termination of protein translation, therefore leading to *MED13L* haploinsufficiency syndrome.

## 1. Introduction

Although techniques for detecting variants are now becoming a routine procedure all over the world, the key question concerns the clinical significance of variants [1]. According to ClinVar data, approximately 12% of submitted DNA sequence variants and copy number variants (CNVs) are pathogenic and about 17% are benign. The significance of the remaining genetic variants is unknown or not yet confirmed. The challenge of performing a detailed analysis and determining the function of the altered gene is therefore still open [2,3]. In order to describe the pathogenicity of DNA sequence variants and/or CNVs, it is necessary to perform a comprehensive molecular and functional genome analysis that involves different fields of study, such as genomics, epigenomics, transcriptomics, proteomics, or even interactomics [4,5].

Some recent studies demonstrated that the pathogenic variants in the *MED13L* (MIM #608771) gene, which is located on chromosome 12 (12q24.21), can be associated with Cornelia de Lange syndrome (CdLS) or CdLS-like phenotypes [6,7]. However, pathogenic heterozygous *MED13L* variants or CNVs mostly cause *MED13L* haploinsufficiency syndrome, which is also defined as impaired intellectual development and distinctive facial features with or without cardiac defects (MRFACD, MIM #616789, ORPHA:369891) [8]. This autosomal dominant neurodevelopmental disorder is characterised by intellectual disability, psychomotor development delay, poor speech, distinctive dysmorphic facial features, and macrostomia. There is also some evidence that various congenital heart defects, including dextro-looped transposition of the great arteries (D-TGA, MIM #608808), can manifest in the affected individuals [8,9]. Less severe clinical phenotypes are supposed to be caused by loss-of-function variants and deletions of the *MED13L* gene rather than missense variants. Different from protein truncating variants or CNVs, missense variants are hypothesised to induce a dominant negative effect in individuals with more severe phenotypes [10]. In fact, the description of pathogenicity mechanisms in *MED13L* haploinsufficiency syndrome is still limited; therefore, a comprehensive analysis of indicated variants is needed. In this study, we evaluated the clinical significance and consequences of a novel heterozygous intragenic *MED13L* deletion through extensive clinical, molecular, and functional characterisations.

## 2. Materials and Methods

### 2.1. Clinical Evaluation of the Individual

The boy, 5 years of age, was one of three children of healthy unrelated Lithuanian parents. He has a healthy older brother and a healthy younger sister. At the birth of the proband, the mother’s age was 31 and the father’s age was 30. The proband was born after a full-term uncomplicated pregnancy via vaginal delivery. At birth, his height was 51 cm (25th–50th centile), his weight was 3200 g (10th centile), and the Apgar scores were 10 at 1 min and 10 at 5 min. After birth, clubfoot was observed and surgical correction was performed at the age of 2 years. Visual impairment and strabismus were also diagnosed at 2 years of age. Although his gross motor development was normal (the boy could sit independently at the age of 5 months and walk at the age of 11 months), his language development was delayed. Psychomotor development was examined at the age of 4.5 years and a mixed specific developmental disorder was diagnosed. Half a year later, the boy was examined by a neurologist, and an EEG, which showed normal results, was performed. The cardiac evaluation revealed no congenital cardiac defects. The hearing of the proband was normal. During an examination at the age of 5 years and 1 month, his head circumference was 48.5 cm (<3rd centile), his height was 107.5 cm (10th–25th centile), and his weight was 18.5 kg (25th centile). A physical examination identified plagiocephaly, strabismus, peculiarities of dermatoglyphics, and a scar after surgery on his right foot. At the time of the examination, the boy could string together 5–6 words to form a sentence, but his use of language was irregular. He had difficulties concentrating and focusing his attention. The boy was not fussy about food and had a good appetite.

### 2.2. DNA Extraction and Single-Nucleotide Polymorphism–Comparative Genomic Hybridisation (SNP-CGH)

With a clinical suspicion of a *MED13L*-related disorder, a detailed molecular and functional analysis was performed. DNA was isolated from the proband’s peripheral blood using the phenol–chloroform–isoamyl alcohol method [11]. A whole-genome SNP-CGH analysis was performed for the proband to detect CNVs using the Human-CytoSNP-12v2.1 BeadChip according to the protocol by Illumina (Illumina, Inc., San Diego, CA, USA) on an Illumina HiScanSQ scanner (Genotyping Module v1.9). GenomeStudio software (Illumina, Inc., San Diego, CA, USA) was used for data processing and primary analysis. QuantiSNP v2.1 software was used for determining CNVs [12]. The data were analysed using GRCh37/hg19 annotation. The results of the SNP-CGH were confirmed via quantitative polymerase chain reaction (qPCR), as described previously [13].

### 2.3. Cell Cultures

A primary fibroblast cell line was obtained from a skin biopsy from the proband and the control individual (*n* = 1). The cell line was cultured in AmnioMAX C-100 Basal Medium (Thermo Fisher Scientific, Waltham, MA, USA) which was supplemented with AmnioMAX C-100 Supplement (Thermo Fisher Scientific, Waltham, MA, USA) and Amphotericin B (Gibco, Waltham, MA, USA) according to the laboratory procedures for human fibroblast cultures [14]. The final cell pellets were subsequently used for gene-expression and gene-editing assays.

### 2.4. RNA Extraction and Reverse Transcription Polymerase Chain Reaction (RT-PCR)

To verify the precise breakpoints of the intragenic *MED13L* gene deletion, the proband’s and control individuals’ (*n* = 3) total blood RNAs were isolated from peripheral blood using a Tempus™ Blood RNA Tube and Tempus™ Spin RNA Isolation Kit (Thermo Fisher Scientific, Life Technologies Corporation, Austin, TX, USA) according to the manufacturer’s protocol and their quality standards. Total RNA was isolated from the proband’s and control individual’s (*n* = 1) fibroblast cell line using an RNeasy Mini Kit (Qiagen, Valencia, CA, USA) for further functional analysis. Complementary DNA (cDNA) was synthesised from total RNA via RT-PCR using a High-Capacity RNA-to-cDNA Kit (Thermo Fisher Scientific Baltics, Vilnius, Lithuania) following the manufacturer’s recommendations and quality standards.

### 2.5. Gene Expression Analysis via Quantitative Polymerase Chain Reaction (qPCR)

The *MED13L* gene expression assays of the proband and control individuals (*n* = 3) were performed via qPCR. The qPCR mix was prepared using a TaqMan Gene Expression Master Mix (Thermo Fisher Scientific Baltics, Vilnius, Lithuania) and TaqMan Gene Expression Assays (Thermo Fisher Scientific, Life Technologies Corporation, Pleasanton, CA, USA), which were designed for the junction of exons 3 and 4 (Hs01573430_m1, Thermo Fisher Scientific) and the junction of exons 16 and 17 (Hs01011103_m1, Thermo Fisher Scientific) of the *MED13L* gene. Primers and a probe for the junction of exons 1 and 2 (Supplementary Table S1) were designed using the Primer-Blast (NCBI) and Custom TaqMan Probes tool (Thermo Fisher Scientific Baltics, Vilnius, Lithuania). The data averaged over the three technical replicates were analysed using SDS v.2.3(Applied Biosystems, Foster City, CA, USA), ExpressionSuite v.1.1 (Applied Biosystems, Foster City, CA, USA), and qbase+ software (Biogazelle, Gent, Belgium). The fold change (FC) of three different locations of the target *MED13L* gene was calculated using the 2^−ΔΔCT^ method and normalised to the *ACTB* (Hs99999903_m1, MIM #102630, Thermo Fisher Scientific, Vilnius, Lithuania) and *GAPDH* (Hs99999905_m1, MIM #138400, Thermo Fisher Scientific, Vilnius, Lithuania) housekeeping genes. The sample from the proband was compared between different locations (the junction of exons 1 and 2, as well as the junction of exons 16 and 17) of the *MED13L* gene (internal control), as well as the samples from three random unrelated healthy individuals (external control). A more than a twofold increase or decrease in the expression level of the *MED13L* gene was considered up- or downregulation, respectively.

### 2.6. Sanger Sequencing of Proband’s cDNA Sample

To elucidate the pathogenicity of the CNV detected, molecular analysis using the proband’s cDNA was performed. The exons from 2 to 15 of the *MED13L* gene were amplified via PCR with specific primers (Supplementary Table S1), which were designed using Primer-Blast (NCBI). PCR products were fractioned using agarose gel electrophoresis and later sequenced using the BigDye Terminator v.3.1 Cycle Sequencing Kit (Thermo Fisher Scientific Baltics, Vilnius, Lithuania) and ABI3130xl Genetic Analyser (Applied Biosystems, Foster City, CA, USA). Sanger sequencing results were analysed with Sequence Analysis v.5.1 (Applied Biosystems, Foster City, CA, USA) and Chromas v.2.4.4 (software (Technelysium, QLD, Australia). The reference sequence of the *MED13L* gene (NCBI: NM_015335.5) was used for the alignment of the obtained sequences. Criteria provided by the American College of Medical Genetics and Genomics (ACMG) were used to assess the pathogenicity of the detected variants [15].

### 2.7. Western Blot

The intragenic *MED13L* deletion was also assessed at the protein level via Western blot analysis. Detached fibroblasts were lysed on ice in a RIPA buffer (Sigma Aldrich, Saint Louis, MO, USA; 1 mL buffer for 10^7^ cells) and an appropriate amount of Halt™ Protease Inhibitor Cocktail (Thermo Fisher Scientific, Waltham, MA, USA) and RNase-free DNase I (Thermo Fisher Scientific, Waltham, MA, USA). After incubation, cell lysates were centrifuged and the protein concentration in the collected supernatant was determined using a Pierce™ BCA Protein Assay Kit (Thermo Fisher Scientific, Waltham, MA, USA). Protein samples were fractioned in 10% SDS-PAGE at 120 V. Semi-dry blotting was applied to transfer proteins on a nitrocellulose membrane (Thermo Fisher Scientific, Waltham, MA, USA). Blots were probed with the anti-MED13L antibody (A302–420A, Bethyl Laboratories, Inc., Montgomery, AL, USA) and anti-β-actin antibody (ab8227, Abcam, UK) for the detection of β-actin as a loading control. Membrane-bound primary antibodies of MED13L and β-actin were detected using a horseradish-peroxidase-conjugated secondary anti-rabbit antibody (31460, Thermo Fisher Scientific, Waltham, MA, USA) and Pierce™ ECL Western Blotting Substrate (Thermo Fisher Scientific, Waltham, MA, USA) chemiluminescence reagent. The signal of MED13L in the samples of the proband and control individuals was normalised according to the signal of β-actin.

### 2.8. MED13L Editing via CRISPR-Cas9 Technique

CRISPR-Cas9 genome editing technology was used to knock out the *MED13L* gene of the control individual (*n* = 1) in a culture of fibroblasts. A gene-editing experiment was performed using no. CRISPR927871_SGM (TrueGuide, Invitrogen, Waltham, MA, USA), Cas9 protein (TrueCut, Invitrogen, Waltham, MA, USA), and a transfection reagent (Lipofectamine, CRISPRMAX, Invitrogen, Waltham, MA, USA) according to the optimised protocols of the manufacturer. To detect the locus-specific cleavage of gDNA, a GeneArt Genomic Cleavage Detection Kit (Invitrogen, Waltham, MA, USA) was used according to the manufacturer’s protocol. PCR products were analysed using agarose gel electrophoresis. ImageJ v.1.52t (National Institutes of Health, Bethesda, MD, USA) gel analysis software was used to determine the relative proportion of DNA contained in each band. The following equation was used to calculate the cleavage efficiency:

Cleavage Efficiency = 1 – ((1 – fraction cleaved)^½^)

For further functional assays, single-cell clones were derived by limiting dilution cloning using ten 96-well plates. RNA extraction, cDNA synthesis, and Sanger sequencing were performed as indicated above. After confirmation of heterozygous *MED13L* variants in the modified cell cultures, functional assays were performed.

### 2.9. Functional Assays of Fibroblast Cultures

After genome editing via the CRISPR-Cas9 experiment, the *MED13L*, *RB1* (Hs00153108_m1), *E2F1* (Hs00153451_m1), and *CCNC* (Hs01029307_m1) gene expression assays were performed using the sample of the proband and the edited sample of the control individual (*n* = 1). The gene expression assay was performed as indicated above. The samples were compared with the internal control (the junction of exons 1 and 2) of the *MED13L* gene.

Cell morphology was determined using an Olympus CKX41 (Olympus Life Science, Tokyo, Japan) inverted phase contrast microscope. Image analysis was performed on early (~3rd–4th) and late (~10th–15th) passages using a JuLITM (NanoEnTek, Seoul, South Korea) analyser.

The viability of the cultured fibroblasts was assessed using Trypan Blue Solution (Gibco) and a Bürker counting chamber (Heinz-Herenz, Hamburg, Germany) according to the manufacturers’ instructions.

The senescence of the cultured fibroblasts was investigated using a Senescence Cells Histochemical Staining Kit (Sigma-Aldrich, St. Louis, MO, USA) according to the manufacturer’s protocol. At the end of the staining procedure, ten pictures were taken in random areas from each specimen. The percentage of senescent cells was calculated using the following formula:

$$\mathrm{Percentage}\;\mathrm{of}\;\mathrm{senescent}\;\mathrm{cells}=\frac{\text{Number of cells with intracellular blue deposits}}{\text{Total number of cells}}\times 100\%$$

### 2.10. In Silico Analysis

The possible effect of the intragenic *MED13L* deletion on protein structure and function was predicted via in silico analysis using a MutationTaster [16], the ExPASy Bioinformatics Resource Portal [17], and the Pfam 32.0 database [18]. In addition, a detailed analysis of various databases (e.g., dbVAR, DGV, and DECIPHER) and a review of scientific literature were performed.

## 3. Results

After comprehensive genetic assays and a detailed in silico analysis, a *de novo* 97.88 kb intragenic deletion in cytoband 12q24.21, which includes exon 3 and 4 of the *MED13L* gene, was detected through SNP-CGH analysis (DECIPHER#430183). According to the data of this analysis, the centromeric breakpoint was located within an 85,018 bp interval delimited using two SNP probes located in the *MED13L* gene: SNP probe rs1895624 (intron 4–5) was deleted, whereas SNP probe rs17426239 (intron 14–15) was not deleted. The telomeric breakpoint was located within a 97,768 bp region between SNP probe rs4145166 (in the *MED13L* gene intron 2–3, deleted) and SNP probe rs3851644 (outside *MED13L*, not deleted). The genomic coordinates of the deletion in chromosome 12 were between 116,523,305 bp and 116,621,185 bp (GRCh37 [hg19/2009]). The minimum and maximum sizes of the deleted region were 97,880 bp and 280,666 bp, respectively. This CNV was confirmed via qPCR.

**Figure 1:**
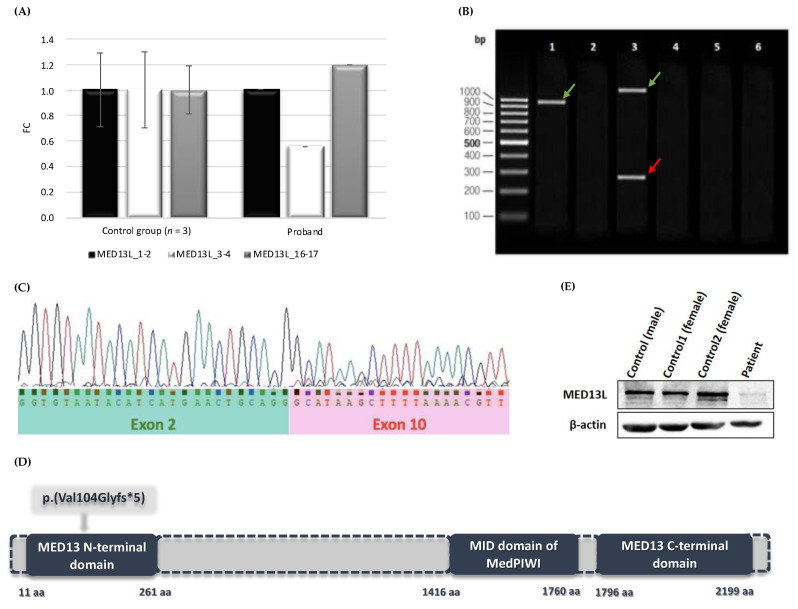
(**A**) *MED13L* gene expression analysis. The results indicate a mean fold change (FC) in target gene expression values of the control group (*n* = 3) versus our proband at three different locations along MED13L: exons 1–2 (marked as MED13L_1-2), exons 3–4 (MED13L_3-4), and exons 16–17 (MED13L_16-17). (**B**) Schematic representation of the gel electrophoresis of the proband’s cDNA synthesised from the RNA sample. Lanes 1, 3, and 5 show the results corresponding to amplicons #A, #B, and #C, respectively. Lanes 2, 4, and 6 correspond to PCR reaction products of the negative control. The green arrows indicate the wild-type alleles and the red arrow indicates the allele with the intragenic *MED13L* deletion. (**C**) The chromatogram represents the Sanger sequencing results of the *MED13L* coding sequence. (**D**) Model of the MED13L protein (UniProtKB: Q71F56) and its functional domains. (**E**) Results of the Western blot analysis.

Further quantitative expression analysis of different *MED13L* exons disclosed the deletion of exons 3–4 and excluded the deletion of exons 1–2 and 16–17: the expression of exons 3–4 of *MED13L* was approximately twofold less than the samples of three random unrelated healthy individuals, while the expression levels of exons 1–2 and exons 16–17 were similar and in a normal range (Figure 1A).

The size of this deletion was later specified via Sanger sequencing. For this purpose, different primer sets listed in Supplementary Table S1 were designed. In the case of a wild-type allele, the expected amplification fragment size of the primer pairs spanning exons (1) from 2 to 8 was 953 nt in length (amplicon #A), (2) from 2 to 10 was 1262 nt (amplicon #B), and (3) from 2 to 15 was 2524 nt (amplicon #C). The results of the gel electrophoresis identified an amplification product of approximately 953 nt (amplicon #A), thus confirming the successful amplification of the proband’s cDNA and suggesting the amplification of only a wild-type allele.

The analysis of the longer amplicon #B disclosed two bands that were approximately 1262 nt and 292 nt in length, thus corresponding to the wild-type and mutated alleles, respectively. The agarose gel electrophoresis of amplicon #C did not fraction any PCR product, thus suggesting that the expected products of both the wild-type and mutated alleles were too long (Figure 1B). To verify these findings, the PCR products of the proband’s cDNA sample were analysed via Sanger sequencing. The analysis of the *MED13L* coding sequence revealed the deletion, which spanned from exons 3 to 10 (Figure 1C). According to the ACMG guideline, this CNV can be classified as pathogenic (e.g., 1A, 2E, 3A, 4B, and 5B)

In silico, NG_023366.1(NM_015335.5):c.(310+1_311-1)_(1280+1_1281-1)del was predicted to result in a truncated MED13L (UniProtKB Q71F56) protein, in which the Med13_N domain is partly altered and the MedPIWI and Med13_C domains are lost (Figure 1D). 

The Western blot method was used to evaluate the expression level of the MED13L protein in fibroblast cell lines from the affected individual and controls. The results showed an approximately fivefold lower level of MED13L in the sample of the proband (relative protein level 0.187) compared with healthy controls (*n* = 3; average relative protein level 0.919) (Figure 1E).

CRISPR-Cas9 genome editing technology was used to knock out the *MED13L* gene of the healthy individual in a culture of skin fibroblasts. The cleavage efficiency was approximately 44%. For further functional assays, single-cell clones were derived after cloning by limiting the dilution procedure. Functional analysis was performed on 11 fibroblast cultures.

Sanger sequencing of PCR products from the modified fibroblast clones of the control individual was performed to identify *MED13L* variants. Different *MED13L* variants were detected in 4 out of 11 fibroblast clones (Table 1). A heterozygous deletion of the second exon was found in three fibroblast clones (#A–#C; Table 1), and a heterozygous deletion of two G nucleotides was identified in one cell culture (#D; Table 1). All these genetic variants were predicted in silico to cause a frameshift and a premature termination codon, as in the case of our proband.

**Figure 2:**
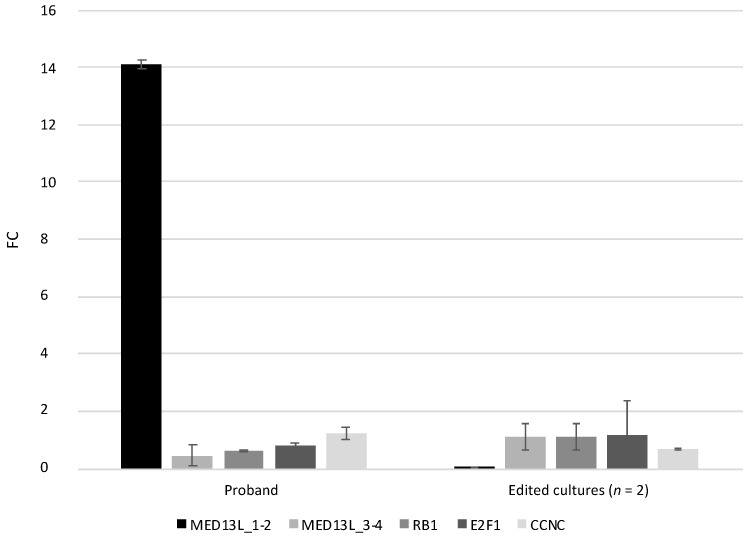
The *MED13L*, *RB1*, *E2F1*, and *CCNC* gene expression analysis. The results indicate a mean fold change in the target gene expression values of the edited fibroblast clones (*n* = 2, #B and #C) of the control individual versus fold change values of the proband’s sample.

The impacts of these genetic variants on the expression of the genes involved in cell cycle regulation—namely, *RB1*, *E2F1*, and *CCNC*—were investigated. For the comparative gene expression analysis, samples from the proband (unmodified fibroblasts) and control individual (edited fibroblasts #B and #C) were compared with the internal control corresponding to a TaqMan probe designed on the junction of exons 1 and 2 of the *MED13L* gene (MED13L_1–2). This probe was chosen as the internal control because, in the aforementioned experiment of the comparative *MED13L* expression, it was found that its expression level was normal in samples of the proband and three control individuals (Figure 1A). qPCR analysis revealed that the expression levels of the junction of exons 3 and 4 of *MED13L, RB1*, *E2F1*, and *CCNC* were, respectively, 31.3-, 22.3-, 17.6-, and 11.6-fold lower in fibroblasts of the proband. Similarly, the expression levels of these genes were, respectively, 12.8-, 12.9-, 11.7-, and 19.8-fold lower in the cells of the control individual after genome editing (Figure 2).

Prior to the genome editing experiment, the viabilities of the control individual’s and proband’s fibroblast cultures were 96% and 89%, respectively. In addition, in the modified fibroblast culture with a heterozygous *MED13L* variant, approximately two times more dead cells were found compared with a fibroblast culture of the same individual without the *MED13L* variant.

**Figure 3:**
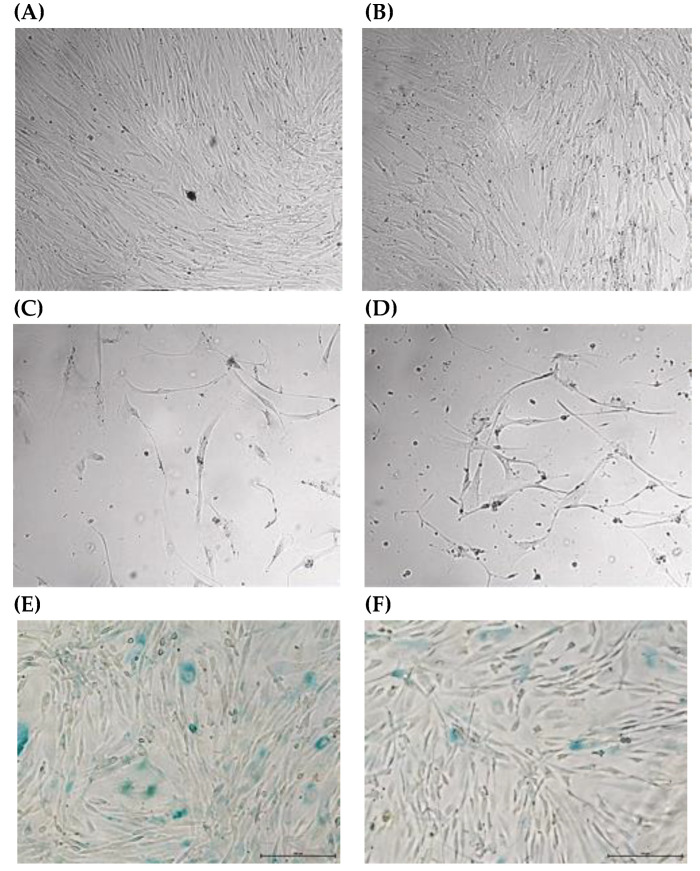
(**A**–**D**) Morphology of cultured fibroblasts (original magnification ×4, scale of 500 μm): early passages (~3rd–4th) of a fibroblast culture of the proband (**A**) and control individual before the gene-editing experiment (**B**); late passages (~10th–15th) of a fibroblast culture of the proband (**C**) and control individual after the gene-editing experiment (**D**). (**E**,**F**) Senescence of cultured fibroblasts (original magnification ×40, scale of 100 μm): early passages (~3rd–4th) of the fibroblast culture of the proband (**E**) and control individual before the gene-editing experiment (**F**).

Cultured fibroblasts of both the proband and control individual were observed microscopically at every passage. Before the gene-editing experiment, adherent long flat spindle-shaped fibroblasts were detected in both cultures at early passages (3rd–4th; Figure 3A,B). The proportion of obviously enlarged fibroblasts with an altered morphology also later increased in both cultures (Figure 3C,D).

The senescence assay showed almost threefold more aging cells of the proband compared with unmodified fibroblasts of the control individual (Figure 3E,F). Furthermore, when long-term fibroblast cultures of the control individual were compared after a CRISPR-Cas9 genome editing experiment, it was found that the fibroblast culture in which *MED13L* variants were not detected contained approximately 55.2% aging cells, while the fibroblast culture in which the heterozygous *MED13L* variant was found was composed of more aging fibroblasts (76.8%).

## 4. Discussion

Ever since the human genome was fully sequenced, the focus of attention has shifted towards the functional characterisation of DNA sequence variants and CNVs, including those affecting gene expression. Recent studies showed that heterozygous variants or CNVs of *MED13L*, which are involved in the regulation of gene expression, result in *MED13L* haploinsufficiency syndrome [8,9,19].

Almost 100 individuals with suspected or confirmed *MED13L* haploinsufficiency syndrome have been reported to date [20,21,22,23,24,25,26,27]. According to the DECIPHER database, there are additional pathogenic *MED13L* deletions and duplications. Based on this database and literature, *MED13L* haploinsufficiency syndrome is characterised by a wide spectrum of clinical features, but almost all individuals, including our proband, have ID, psychomotor developmental delay, and speech delay. The prevalence of other phenotypic features, such as muscle hypotonia, brain anomalies, craniofacial deformity, ophthalmological defects, and anomalies of the hands and/or feet, are lower (20.8–80.5%). The congenital heart defects, previously highlighted as one of the main features of *MED13L* haploinsufficiency syndrome, were diagnosed in only 15 (20.8%) individuals (Supplementary Table S2).

The protein encoded by the *MED13L* gene is the Mediator Complex Subunit 13-Like (UniProtKB Q71F56), also known as the Thyroid Hormone Receptor-Associated Protein 2 (THRAP2). MED13L, together with 29 other subunits, forms a large multiprotein complex called the mediator complex in humans. Structurally, the mediator complex, which is highly conserved in all eukaryotes, is divided into four modules: the head, middle, tail, and CDK8 kinase modules. The latter module contains CDK8, cyclin C, MED12 (or MED12L), and MED13 (or MED13L) subunits. Recent studies indicated that MedPIWI, which is the core globular domain of the MED13 or MED13L protein, is predicted to trigger the conformational switch in the CDK8 subunit that regulates the mediator complex. Zhang et al. (2020), by silencing MED13L in non-small-cell lung cancer cells, showed disrupted interaction between the CDK8 kinase module and the core mediator. Meanwhile, the head module, together with the middle module, plays an essential role during the assembly of the pre-initiation complex by contacting the RNA polymerase type II, thus stabilising its interaction with the DNA-binding transcription factors. Therefore, the mediator complex, also known as a transcriptional co-activator, acts as a physical and functional bridge between both general transcription factors and basal transcription machinery to ensure accurate gene expression regulation [28,29,30,31,32,33,34]. Moreover, the mediator complex is essential for RNA elongation, transcriptional termination, alternative splicing, and chromatin remodeling processes [35].

According to GTEx [36], *MED13L* is widely expressed in almost all human tissues. High expression of this gene is detected in the uterus, arteries, adipose tissue, brain structures, breast, and cultured fibroblasts. It plays an essential role in the early development of the heart, brain, and central nervous system [9,37,38]. The importance of this gene in the development of the central nervous system was demonstrated in recent studies reported by Hamada et al. (2021 and 2023). It was shown that MED13L is localised not only in the cell nucleus but also in synapses, thus suggesting that MED13L has an important impact on synaptic functions [37]. Furthermore, it was shown that pathogenic *MED13L* variants trigger the dendritic development of cerebral cortical neurons in the mammalian brain [38]. Utami et al. (2014) found that morpholino-mediated knockdown of *med13b*, which is the zebrafish closest orthologue of *MED13L*, results in anomalies of cartilage structure due to defects in the early migration of cranial neural crest cells (NCCs). This knockdown zebrafish model partly phenocopies craniofacial abnormalities observed in humans [39]. Recent transcriptional analysis of fibroblasts with pathogenic *MED13L* variants revealed reduced mRNA levels in several genes that ensure appropriate mitochondrial function [40]. Moreover, microarray analysis of *MED13L*-knockdown neurons derived from human embryonic stem cells (hESCs) revealed significant differences in the expression of 1117 genes that are mostly involved in Wnt and FGF signalling pathways [39]. There is some evidence that *MED13L* is a functional Rb/E2F co-factor [41]. At the molecular level, Rb/E2F signalling pathways regulate the transition from G1 to S phase. The retinoblastoma (Rb) tumour suppressor directly binds to the transactivation domain of E2F and suppresses its function. The phosphorylation of Rb, which is initiated by cyclin D/CDK4 complexes, disrupts Rb/E2F interactions. Therefore, the active form of E2F initiates the transcription of cell cycle genes (e.g., *CCNA2* coding cyclin A) [42]. A previous study showed that *MED13L* contributes to the Rb/E2F-mediated inhibition of cell proliferation, repression of cell cycle target genes, and control of cell growth [41].

In this study, a novel intragenic *MED13L* deletion, namely, NG_023366.1(NM_015335.5):c.(310+1_311-1)_(1280+1_1281-1)del, was detected in a proband with clinical features of *MED13L* haploinsufficiency syndrome. In silico analysis disclosed that this deletion results in a translational frameshift and formation of premature termination codon (NP_056150.1:p.(Val104Glyfs*5). At the protein level, these changes were predicted to result in protein truncation or haploinsufficiency due to mRNA degradation induced by nonsense-mediated decay (NMD). If NMD affects almost all mRNA transcripts, protein haploinsufficiency is the leading molecular mechanism of pathogenesis. On the other hand, the production of a truncated protein because of complete NMD avoidance can significantly impact protein function due to an altered protein structure [43]. The NMD pathway triggers the degradation of mRNAs harbouring a premature termination codon that is located more than 200 nt downstream of the start codon and more than 50–55 nt upstream of the last exon–exon junction [44,45]. In our study, the premature termination codon of the *MED13L* gene is sufficiently distant from the start codon and the last exon–exon junction. This region could therefore be recognised by the NMD. This assumption was confirmed via Western blotting, which showed significantly decreased expression of the MED13L protein in fibroblast cell lines from the proband compared with control individuals.

Even though the disrupted Wnt, FGF, and Rb/E2F signalling pathways due to pathogenic DNA sequence variants/CNVs of the *MED13L* gene are thought to be the leading pathogenic mechanism in *MED13L* haploinsufficiency syndrome, the fundamental molecular mechanism underlying the pathogenesis of these genetic alterations has not yet been fully understood [19,39,41]. To confirm our findings at the human cellular level, CRISPR-Cas9 technology was used to knock down the *MED13L* gene in a culture of skin fibroblasts of the healthy individual. Heterozygous *MED13L* variants were detected in four of the eleven modified fibroblast clones (#A–#D, Table 1). No homozygous variants of *MED13L* were identified. During the cultivation of the edited fibroblast clones, the morphology of a few cultures obviously changed earlier, and decreased cell proliferation and viability were noted. For these reasons, it was impossible to expand these cells to a sufficient number for further functional assays. This could be explained by the presence of the homozygous *MED13L* variants that are possibly lethal to the cell. To confirm this assumption, another research strategy, such as DNA extraction from a single cell immediately after genome editing, is suggested. Nevertheless, in our study, all heterozygous *MED13L* variants in silico were predicted to cause a frameshift and premature termination codon, which resulted in *MED13L* haploinsufficiency. Therefore, the proband’s genotype was successfully reproduced at the cellular level.

In order to evaluate the functional impact of these genetic variants, a comparative gene expression analysis of *MED13L*, *RB1*, *E2F1*, and *CCNC* was performed. This analysis revealed that the expression levels of the junction of exons 1 and 2 of the *MED13L* gene and the *RB1*, *E2F1*, and *CCNC* genes were from 11.7- to 31.3-fold lower in fibroblasts of both the proband and control individual after the genome editing. According to GTEx data, the expression levels of these genes (*RB1* TPM = 38.1, *E2F1* TPM = 9.1, *CCNC* TPM = 29.8) in cultured fibroblasts are similar to the expression level of *MED13L* (TPM = 28.9). The expression of *RB1* is 1.3-fold higher, while the expressions of *E2F1* and *CCNC* are 3.2- and 0.99-fold lower than the expression level of the *MED13L* gene in the samples of cultured fibroblasts. However, in our study, the qPCR analysis revealed a significant reduction in the expression level of *RB1*, *E2F1*, and *CCNC* in fibroblasts of both the proband and the control individual, whose genome was edited via CRISPR-Cas9 technology.

Furthermore, cultured fibroblasts of the proband and control individuals (both modified and unmodified) were observed microscopically at every passage and no significant difference in morphology was observed between the cell cultures. Nevertheless, in a modified fibroblast culture with a heterozygous *MED13L* variant, approximately two times more dead cells were found than in the fibroblast culture without the *MED13L* variant. Moreover, by staining the cells for SA-β-gal (senescence-associated beta-galactosidase), which is known as the most widely used biomarker for senescent cells [46], we confirmed almost three times more aging cells in the fibroblast culture of our proband compared with the unmodified fibroblasts of the control individual. Hence, the *MED13L* gene-editing experiment provided additional evidence that heterozygous *MED13L* changes led to a reduction in cell viability, acceleration of the aging process, and inhibition of the expression of certain genes.

## 5. Conclusions

In conclusion, the clinical, molecular, and functional characterisation of intragenic *MED13L* deletion and successfully managed genome editing via CRISPR-Cas9 technology in this study provided additional evidence to comprehend the aetiology and pathophysiology of *MED13L* haploinsufficiency syndrome. This fundamental scientific knowledge, together with the expansion of the capabilities of molecular diagnostic and functional investigations, may contribute to the development of novel diagnostic and therapeutic strategies in the future. A straightforward benefit of the results that were obtained is a molecular diagnosis, which was given to the family and provided an opportunity to improve clinical care and follow-up.

## Figures and Tables

**Table 1:** *MED13L* variants detected using Sanger sequencing after genome editing via CRISPR-Cas9 technology.

Cell Culture	Genotype	*MED13L* Variants Caused by CRISPR-Cas9	In Silico Predicted Effect at the Protein Level
#A	Heterozygous	NG_023366.1(NM_015335.5):c.(72+1_73-1)_(310+1_311-1)del	NP_056150.1:p.(Ala25Cysfs*13)
#B	Heterozygous	NG_023366.1(NM_015335.5):c.(72+1_73-1)_(310+1_311-1)del	NP_056150.1:p.(Ala25Cysfs*13)
#C	Heterozygous	NG_023366.1(NM_015335.5):c.(72+1_73-1)_(310+1_311-1)del	NP_056150.1:p.(Ala25Cysfs*13)
#D	Heterozygous	NM_015335.5:c.95_96del	NP_056150.1:p.(Trp32Serfs*1)

## Data Availability

The main data generated and analysed during this study are included in this article and its supplementary information files. Any additional information is available from the authors upon request.

## Supplementary Materials

The following supporting information can be downloaded from https://www.mdpi.com/article/10.3390/medicina59071225/s1: Table S1. The conditions for PCR amplification of the *MED13L* gene in gDNA and cDNA samples: primer sequences, location, annealing temperature, and lengths of the amplicons; Table S2. Clinical features of individuals with pathogenic DNA sequence variants or CNVs of the *MED13L* gene.

## Abbreviations

ACMG	American College of Medical Genetics and Genomics
CdLS	Cornelia de Lange syndrome
cDNA	Complementary DNA
CNVs	Copy number variants
CRISPR-Cas9	Clustered regularly interspaced short palindromic repeats/CRISPR-associated protein 9
D-TGA	Dextro-looped transposition of the great arteries
EEG	Electroencephalography
FC	Fold change
gDNA	Genomic DNA
kb	Kilobase
*MED13L*	Mediator Complex Subunit 13-Like
MRFACD	Impaired intellectual development and distinctive facial features with or without cardiac defects
NCCs	Cranial neural crest cells
NMD	Nonsense-mediated decay
nt	Nucleotide
qPCR	Quantitative polymerase chain reaction
Rb	Retinoblastoma
SA-β-gal	Senescence-associated beta-galactosidase
SNP-CGH	Single Nucleotide Polymorphism array and Comparative Genomic Hybridisation array
SNP	Single Nucleotide Polymorphism
THRAP2	Thyroid Hormone Receptor-Associated Protein 2

## References

[1] Rehm H.L., Fowler D.M. (2020). Keeping up with the genomes: Scaling genomic variant interpretation. Genome Med..

[2] Bell C.J., Dinwiddie D.L., Miller N.A., Hateley S.L., Ganusova E.E., Mudge J., Langley R.J., Zhang L., Lee C.C., Schilkey F.D. (2011). Carrier Testing for Severe Childhood Recessive Diseases by Next-Generation Sequencing. Sci. Transl. Med..

[3] Kircher M., Kelso J. (2010). High-throughput DNA sequencing-concepts and limitations. Bioessays.

[4] Sun Y.V., Hu Y.-J. (2016). Integrative Analysis of Multi-omics Data for Discovery and Functional Studies of Complex Human Diseases. Adv. Genet..

[5] Bunnik E.M., Le Roch K.G. (2013). An Introduction to Functional Genomics and Systems Biology. Adv. Wound Care.

[6] Aoi H., Mizuguchi T., Ceroni J.R., Kim V.E.H., Furquim I., Honjo R.S., Iwaki T., Suzuki T., Sekiguchi F., Uchiyama Y. (2019). Comprehensive genetic analysis of 57 families with clinically suspected Cornelia de Lange syndrome. J. Hum. Genet..

[7] García-Gutiérrez P., García-Domínguez M. (2021). BETting on a Transcriptional Deficit as the Main Cause for Cornelia de Lange Syndrome. Front. Mol. Biosci..

[8] Adegbola A., Musante L., Callewaert B., Maciel P., Hu H., Isidor B., Picker-Minh S., Le Caignec C., Chiaie B.D., Vanakker O. (2015). Redefining the MED13L syndrome. Eur. J. Hum. Genet..

[9] Muncke N., Jung C., Rüdiger H., Ulmer H., Roeth R., Hubert A., Goldmuntz E., Driscoll D., Goodship J., Schön K. (2003). Missense Mutations and Gene Interruption in PROSIT240, a Novel TRAP240 -like Gene, in Patients with Congenital Heart Defect (Transposition of the Great Arteries). Circulation.

[10] Smol T., Petit F., Piton A., Keren B., Sanlaville D., Afenjar A., Baker S., Bedoukian E.C., Bhoj E.J., Bonneau D. (2018). MED13L-related intellectual disability: Involvement of missense variants and delineation of the phenotype. Neurogenetics.

[11] Javadi A., Shamaei M., Ziazi L.M., Pourabdollah M., Dorudinia A., Seyedmehdi S.M., Karimi S. (2014). Qualification Study of Two Genomic DNA Extraction Methods in Different Clinical Samples. Tanaffos.

[12] Colella S., Yau C., Taylor J.M., Mirza G., Butler H., Clouston P., Bassett A.S., Seller A., Holmes C.C., Ragoussis J. (2007). QuantiSNP: An Objective Bayes Hidden-Markov Model to detect and accurately map copy number variation using SNP genotyping data. Nucleic Acids Res..

[13] Preikšaitienė E., Ambrozaitytė L., Maldžienė Z., Morkūnienė A., Cimbalistienė L., Rančelis T., Utkus A., Kučinskas V. (2016). Identification of genetic causes of congenital neurodevelopmental disorders using genome wide molecular technologies. Acta Med. Litu..

[14] Villegas J., McPhaul M. (2005). Establishment and Culture of Human Skin Fibroblasts. Curr. Protoc. Mol. Biol..

[15] Riggs E.R., Andersen E.F., Cherry A.M., Kantarci S., Kearney H., Patel A., Raca G., Ritter D.I., South S.T., Thorland E.C. (2020). Technical standards for the interpretation and reporting of constitutional copy-number variants: A joint consensus recommendation of the American College of Medical Genetics and Genomics (ACMG) and the Clinical Genome Resource (ClinGen). Genet. Med..

[16] Schwarz J.M., Cooper D.N., Schuelke M., Seelow D. (2014). MutationTaster2: Mutation prediction for the deep-sequencing age. Nat. Methods.

[17] Gasteiger E. (2003). ExPASy: The proteomics server for in-depth protein knowledge and analysis. Nucleic Acids Res..

[18] Finn R.D., Bateman A., Clements J., Coggill P., Eberhardt R.Y., Eddy S.R., Heger A., Hetherington K., Holm L., Mistry J. (2014). Pfam: The protein families database. Nucleic Acids Res..

[19] Asadollahi R., Zweier M., Gogoll L., Schiffmann R., Sticht H., Steindl K., Rauch A. (2017). Genotype-phenotype evaluation of MED13L defects in the light of a novel truncating and a recurrent missense mutation. Eur. J. Med. Genet..

[20] Tørring P.M., Larsen M.J., Brasch-Andersen C., Krogh L.N., Kibæk M., Laulund L., Illum N., Dunkhase-Heinl U., Wiesener A., Popp B. (2019). Is MED13L-related intellectual disability a recognizable syndrome?. Eur. J. Med. Genet..

[21] Yi Z., Zhang Y., Song Z., Pan H., Yang C., Li F., Xue J., Qu Z. (2020). Report of a de novo c.2605C > T (p.Pro869Ser) change in the MED13L gene and review of the literature for MED13L-related intellectual disability. Ital. J. Pediatr..

[22] Romero M.S.J., Carrasco-Salas P., Benítez-Burraco A. (2018). Language and Cognitive Impairment Associated with a Novel p.Cys63Arg Change in the MED13L Transcriptional Regulator. Mol. Syndr..

[23] Carvalho L.M.L., Costa S.S., Campagnari F., Kaufman A., Bertola D.R., Silva I.T., Krepischi A.C.V., Koiffmann C.P., Rosenberg C. (2021). Two novel pathogenic variants in *MED13L*: One familial and one isolated case. J. Intellect. Disabil. Res..

[24] Sabo A., Murdock D., Dugan S., Meng Q., Gingras M., Hu J., Muzny D., Gibbs R. (2020). Community-based recruitment and exome sequencing indicates high diagnostic yield in adults with intellectual disability. Mol. Genet. Genom. Med..

[25] Park S.-J., Lee N., Jeong S.-H., Jeong M.-H., Byun S.-Y., Park K.-H. (2022). Genetic Aspects of Small for Gestational Age Infants Using Targeted-Exome Sequencing and Whole-Exome Sequencing: A Single Center Study. J. Clin. Med..

[26] Bessenyei B., Balogh I., Mokánszki A., Ujfalusi A., Pfundt R., Szakszon K. (2022). *MED13L*-related intellectual disability due to paternal germinal mosaicism. Mol. Case Stud..

[27] Mainali A., Athey T., Bahl S., Hung C., Caluseriu O., Chan A., Eaton A., Ghai S.J., Kannu P., MacPherson M. (2022). Diagnostic yield of clinical exome sequencing in adulthood in medical genetics clinics. Am. J. Med. Genet. Part A.

[28] Burroughs A.M., Iyer L.M., Aravind L. (2013). Two novel PIWI families: Roles in inter-genomic conflicts in bacteria and Mediator-dependent modulation of transcription in eukaryotes. Biol. Direct.

[29] Soutourina J. (2018). Transcription regulation by the Mediator complex. Nat. Rev. Mol. Cell Biol..

[30] Verger A., Monté D., Villeret V. (2019). Twenty years of Mediator complex structural studies. Biochem. Soc. Trans..

[31] Yin J.-W., Wang G. (2014). The Mediator complex: A master coordinator of transcription and cell lineage development. Development.

[32] Napoli C., Schiano C., Soricelli A. (2019). Increasing evidence of pathogenic role of the Mediator (MED) complex in the development of cardiovascular diseases. Biochimie.

[33] Zhang N., Song Y., Xu Y., Liu J., Shen Y., Zhou L., Yu J., Yang M. (2020). MED13L integrates Mediator-regulated epigenetic control into lung cancer radiosensitivity. Theranostics.

[34] Stieg D.C., Cooper K.F., Strich R. (2020). The extent of cyclin C promoter occupancy directs changes in stress-dependent transcription. J. Biol. Chem..

[35] Schiano C., Casamassimi A., Vietri M.T., Rienzo M., Napoli C. (2014). The roles of Mediator complex in cardiovascular diseases. Biochim. Biophys. Acta-Gene Regul. Mech..

[36] Lonsdale J., Thomas J., Salvatore M., Phillips R., Lo E., Shad S., Hasz R., Walters G., Garcia F., Young N. (2013). The Genotype-Tissue Expression (GTEx) project. Nat. Genet..

[37] Hamada N., Iwamoto I., Nishikawa M., Nagata K.-I. (2021). Expression Analyses of Mediator Complex Subunit 13-Like: A Responsible Gene for Neurodevelopmental Disorders during Mouse Brain Development. Dev. Neurosci..

[38] Hamada N., Iwamoto I., Nagata K. (2023). MED13L and its disease-associated variants influence the dendritic development of cerebral cortical neurons in the mammalian brain. J. Neurochem..

[39] Utami K.H., Winata C.L., Hillmer A.M., Aksoy I., Long H.T., Liany H., Yan E.C.G., Mathavan S., Hong S.T.K., Korzh V. (2014). Impaired Development of Neural-Crest Cell Derived Organs and Intellectual Disability Caused by MED13L haploinsufficiency. Hum. Mutat..

[40] Chang K.-T., Jezek J., Campbell A.N., Stieg D.C., Kiss Z.A., Kemper K., Jiang P., Lee H.-O., Kruger W.D., van Hasselt P.M. (2022). Aberrant cyclin C nuclear release induces mitochondrial fragmentation and dysfunction in MED13L syndrome fibroblasts. iScience.

[41] Angus S., Nevins J.R. (2012). A role for Mediator complex subunit MED13L in Rb/E2F-induced growth arrest. Oncogene.

[42] Harbour J.W. (2000). The Rb/E2F pathway: Expanding roles and emerging paradigms. Genes Dev..

[43] Fusco C., Morlino S., Micale L., Ferraris A., Grammatico P., Castori M. (2019). Characterization of Two Novel Intronic Variants Affecting Splicing in FBN1-Related Disorders. Genes.

[44] Hug N., Longman D., Cáceres J.F. (2016). Mechanism and regulation of the nonsense-mediated decay pathway. Nucleic Acids Res..

[45] Raimondeau E., Bufton J.C., Berger-Schaffitzel C.H. (2018). New insights into the interplay between the translation machinery and nonsense-mediated mRNA decay factors. Biochem. Soc. Trans..

[46] Wagner W., Bork S., Lepperdinger G., Joussen S., Ma N., Strunk D., Koch C. (2010). How to track cellular aging of mesenchymal stromal cells?. Aging.

