# Supplementary material for: Molecular and Functional Characterisation of a Novel Intragenic 12q24.21 Deletion Resulting in MED13L Haploinsufficiency Syndrome
Source: Medicina (Kaunas). 2023 Jun 29;59(7):1225. doi: 10.3390/medicina59071225 (PMC10385642; doi:10.3390/medicina59071225)
Supplement: Supplementary file 1 [file medicina-59-01225-s001.zip › medicina-2445400-supplementary.pdf]

Supplementary Table S1. The conditions for PCR amplification of the *MED13L* gene in gDNA and cDNA samples: primer sequences, location, annealing temperature, and lengths of the amplicons.

Gene (RefSeq transcript)	Sample	Primers (5′ → 3′)	Location	Annealing temperature	Amplicon length
***MED13L* (NM_015335.4, NG_023366.1)**	cDNA	**F:** CGGGAATCAAATGGCGTAGG	2 exon	59 ^{0}C	953 bp
		**R:** AGCACTCTGCATACCTCCAC	8 exon	59,5 ^{0}C	
		**F:** CGGGAATCAAATGGCGTAGG	2 exon	59 ^{0}C	1262 bp
		**R:** CCCTGGTTGAGATACTGTGGG	10 exon	59,8 ^{0}C	
		**F:** CGGGAATCAAATGGCGTAGG	2 exon	59 ^{0}C	2524 bp
		**R:** GGTGGAGTGGGAAACATCCT	15 exon	59,3 ^{0}C	
	cDNA (Gene expression assay)	**F:** CGAGCCTGGAGGATTGTCA	1 exon	59,1 ^{0}C	118 bp
		**R:** GCTGGGGCTGAAATTATGGG	2 exon	59 ^{0}C	
		**TaqMan™ probe:** ACCTCTTTTCGCTGGCTGAACTCAC	1–2 exon junction	61,2 ^{0}C	
	gDNA (CRISPR-Cas9 assay)	**F:** CTCCCACCTTAAAATTCGAGAAGA	1 intron	58,5 ^{0}C	559 bp
		**R:** GGGGTGAGAATGTTCCTTTTGAG	2 intron	59,5 ^{0}C	

F – forward primer; R – reverse primer.

Supplementary Table S2. Clinical features of individuals with pathogenic DNA sequence variants or CNVs of the *MED13L* gene.

	Individual	ID	Psychomotor developmental delay	Speech delay	Craniofacial deformity	Anomalies of hands and/or feet	Hypotonia	Ophthalmological anomalies	Cerebral anomalies	Congenital heart defects	Autistic behaviour
	**The proband**	+	+	+	–	+	–	+	–	–	–
	**Literature (n = 79) [20-27]**	77/77	79/79	77/78	58/75	34/74	54/74	27/75	27/60	14/70	18/72
**12q24.21 deletion (DECIPHER)**	#264232	NA	+	NA	+	+	NA	NA	+	NA	NA
	#260719	NA	+	NA	NA	+	NA	+	NA	NA	NA
	#271765	NA	+	NA	+	+	NA	NA	NA	NA	NA
	#271595	+	+	NA	+	NA	NA	NA	NA	NA	+
	#285418	+	NA	NA	NA	NA	NA	NA	NA	NA	NA
	#283932	NA	+	+	+	NA	NA	+	NA	NA	NA
	#277527	NA	+	+	+	NA	NA	+	NA	NA	NA
	#272267	NA	+	NA	+	NA	NA	NA	NA	+	NA
	#296332	NA	+	NA	+	+	+	+	NA	NA	NA
	#328479	NA	+	+	+	NA	NA	NA	NA	NA	NA
	#322949	NA	+	+	+	NA	+	+	NA	NA	NA
	#363856	+	+	+	NA	NA	+	NA	NA	NA	NA
	#307630	NA	+	+	+	+	NA	+	NA	NA	NA
	#401741	+	+	+	NA	NA	NA	+	NA	NA	+
**12q24.21 duplication**	#296364	+	+	+	NA	NA	NA	NA	NA	NA	NA
	#296365	NA	+	NA	+	NA	NA	NA	NA	NA	NA
	#272268	Na	+	+	+	+	NA	NA	NA	NA	NA
	**Total number**	83/83	97/97	87/88	70/87	41/81	57/78	35/83	28/62	15/72	20/75

**Total number (%)**	100 %	100 %	98,9 %	80,5 %	50,6 %	73,1 %	42,2 %	45,2 %	20,8 %	26,7 %

NA - no data available.
